# Secondary cell wall patterning—connecting the dots, pits and helices

**DOI:** 10.1098/rsob.210208

**Published:** 2022-05-04

**Authors:** Huizhen Xu, Alessandro Giannetti, Yuki Sugiyama, Wenna Zheng, René Schneider, Yoichiro Watanabe, Yoshihisa Oda, Staffan Persson

**Affiliations:** ^1^ School of Biosciences, The University of Melbourne, Parkville, Victoria 3010, Australia; ^2^ Department of Plant and Environmental Sciences, University of Copenhagen, 1871 Frederiksberg C, Denmark; ^3^ Copenhagen Plant Science Center, University of Copenhagen, 1871 Frederiksberg C, Denmark; ^4^ The Sainsbury Laboratory, University of Cambridge, Bateman Street, Cambridge CB2 1LR, UK; ^5^ Institute of Biochemistry and Biology, Plant Physiology Department, University of Potsdam, 14476 Potsdam, Germany; ^6^ Institute for Research Initiatives, Nara Institute of Science and Technology, 8916-5 Takayama, Ikoma, Nara 630-0192, Japan; ^7^ Department of Gene Function and Phenomics, National Institute of Genetics, 1111 Yata, Mishima, Shizuoka 411-8540, Japan; ^8^ Department of Genetics, The Graduate University for Advanced Studies, SOKENDAI, 1111 Yata, Mishima, Shizuoka 411-8540, Japan; ^9^ Joint International Research Laboratory of Metabolic and Developmental Sciences, State Key Laboratory of Hybrid Rice, School of Life Sciences and Biotechnology, Shanghai Jiao Tong University, Shanghai, People's Republic of China

**Keywords:** plant cell wall, microtubules, xylem, cell wall patterning, cellulose

## Abstract

All plant cells are encased in primary cell walls that determine plant morphology, but also protect the cells against the environment. Certain cells also produce a secondary wall that supports mechanically demanding processes, such as maintaining plant body stature and water transport inside plants. Both these walls are primarily composed of polysaccharides that are arranged in certain patterns to support cell functions. A key requisite for patterned cell walls is the arrangement of cortical microtubules that may direct the delivery of wall polymers and/or cell wall producing enzymes to certain plasma membrane locations. Microtubules also steer the synthesis of cellulose—the load-bearing structure in cell walls—at the plasma membrane. The organization and behaviour of the microtubule array are thus of fundamental importance to cell wall patterns. These aspects are controlled by the coordinated effort of small GTPases that probably coordinate a Turing's reaction–diffusion mechanism to drive microtubule patterns. Here, we give an overview on how wall patterns form in the water-transporting xylem vessels of plants. We discuss systems that have been used to dissect mechanisms that underpin the xylem wall patterns, emphasizing the VND6 and VND7 inducible systems, and outline challenges that lay ahead in this field.

## Graphical abstract

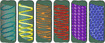

## 1. Introduction

Plant cells are surrounded by cell walls that support plant stature and direct cell expansion, thus determining the physical shape and structure of plants. Plant cell walls consist largely of different types of polysaccharides, proteins, solutes and, in some cases, polyphenolic compounds termed lignin [1]. The polysaccharides are further divided into three main groups; cellulose, hemicelluloses and pectins. Cellulose is a relatively simple polysaccharide that consists of β-1,4-linked glucans, which adhere to each other via hydrogen bonds and van der Waals forces [2]. The resulting cellulose microfibrils contribute to the load-bearing structures of the plant cell wall with Young's modulus (or elastic modulus) of about 115 to 140 GPa [3], or just below that of steel (grade 316 stainless steel ≈190 GPa) [4]. The hemicelluloses consist of several different glycan structures that typically are named according to their backbone and sidechain contents. For example, xyloglucans comprise a glucan backbone with xylose-rich sidechains, xylans have a xylose-based backbone and the backbone of mannans and glucomannans consists of mannose and mannose/glucose residues [5]. Some hemicelluloses engage with the cellulose microfibrils at distinct hydrophobic sites, referred to as mechanical hotspots that contribute to cell wall extensibility or align with the microfibrils to change their chemical characteristics [6,7]. Pectins are a diverse class of charged polysaccharides, comprising a galacturonic acid-containing backbone, and include homogalacturonan, and rhamnogalacturonan I and II [8]. Pectins may connect to cellulose and to hemicelluloses, thereby establishing a strong cross-linked matrix that provides physical strength to the walls and enable cell–cell interactions [9,10].

Plant cell walls are subdivided into three types of walls: the primary and secondary cell walls (SCWs), and the middle lamella. The middle lamella is pectin-rich and deposited during cytokinesis to function as a molecular glue to maintain cell–cell adhesion [11]. While lignification is strongly associated with SCW synthesis, the initiation of lignification can occur at the cell corners and middle lamella prior to spreading to secondary wall thickening layers [12–14]. For instance, the middle lamella in both radiata pine (*Pinus radiata*) and red beech (*Nothofagus fusca*) are highly lignified [15].

Primary walls are deposited after the middle lamella and are flexible poly-lamellate structures that allow for cell expansion and that, together with solute influx into the vacuole, contribute the turgor of the plant cell [16]. These walls largely consist of cellulose, hemicelluloses and pectins that engage with each other through covalent, hydrogen and ionic bonds and forces [1]. Finally, SCWs are produced around cells that need structural support for their functions and are typically synthesized when cells either have stopped growing or are in their final phases of doing so [17]. These walls largely contain cellulose, hemicelluloses and lignin, and are the main focus of this review.

Cellulose microfibrils in SCWs are often deposited as a multilaminar structure and are typically composed of three layers (S1, S2 and S3), which are characterized by distinct cellulose content, crystallinity, degree of polymerization and microfibril orientation and organization [18]. The S1 layer is deposited first to become the outermost layer, with crossed microfibril orientation. The middle S2 layer accounts for about 80% of the SCW thickness [19]. Here, microfibrils are orientated nearly in parallel to the fibre axis while the microfibrils in the inner S3 layer are oriented in a flat helix [15,20]. However, secondary wall structures in reaction wood (i.e. compression wood and tension wood that is a result of bending or tilting of stems and branches) differ from regular wood. Compression wood generally comprises an outer S1 layer, a lignin-rich outer S2 layer termed S2(L), and an inner S2 layer, but lacks the S3 layer entirely [20,21]. Tension wood appears to lack one or more secondary wall layers but contain a thickened gelatinous layer (G-layer), which possesses low lignin but high levels of cellulose [19,20].

Examples of cells/tissues that are associated with SCW synthesis include; interfascicular fibres that interlink the vasculature in many dicot stems, sclereids in some fruits (sometimes termed stone cells or brachysclereids), cotton fibres, seed coats of many plants and in the anther endothecium to provide forces to release pollen grains [22]. Perhaps the most eye-popping SCW-related process occurs during seed dispersal of several members of the *Brassicaceae* family where tension is built through the coordination of SCW deposition and turgor [23]. Nevertheless, the development of xylem vessels is arguably the most well-studied SCW-producing process. Here, the SCWs provide mechanical support and strength to enable xylem to transport water, minerals and nutrients over long distances from the ground tissue to aerial parts of the plant [17,24].

The xylem has a longstanding tradition in plant biology. While the term was first coined by Carl Wilhelm von Nägeli in the mid-1800s, this tissue was observed and understood, at least in part, already in the late seventeenth century by Marcello Malpighi. The xylem tissue contributes a substantial part of the biomass of most plants and has therefore attracted substantial scientific interest, in part driven by its economic importance. While xylem contains several different cell types, intricate SCW patterns are associated with tracheids and vessels, also referred to as tracheary elements (TEs) [17]. TE development is a well-defined process that involves initiation and specification, patterned cell wall deposition and finally programmed cell death. The differentiation and development of TEs, as well as the biosynthesis of different cell wall types, have been extensively reviewed elsewhere [17,24,25]. In this review, we focus on wall patterns associated with the TEs, how different systems, mainly focusing on the recently developed so-called VND systems, have been used to understand wall pattern regulation and on providing an extensive outlook of challenges and questions that need to be addressed in this area.

## 2. Xylem organization and secondary cell wall patterns

**Figure 1.**
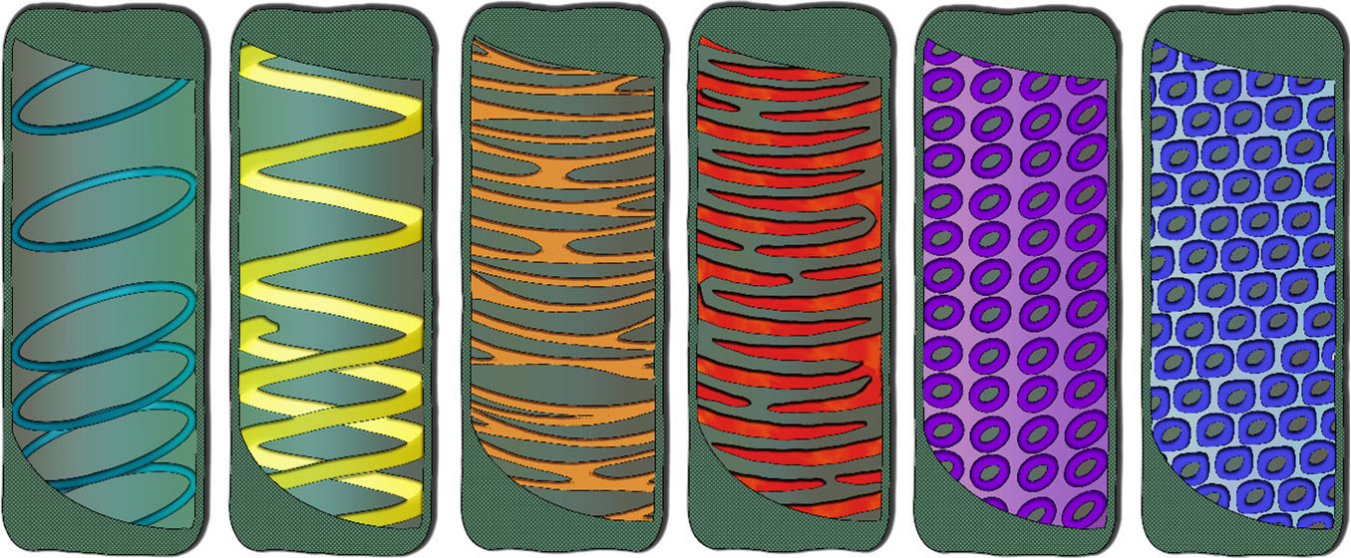
Examples of secondary cell wall pattern types. From left to right: annular (cyan), helical (yellow), reticulate (orange), scalariform (red), opposite-pitted (purple) and alternate-pitted (blue).

Most primary walls are produced in certain patterns to support anisotropic growth, for example around elongating hypocotyl cells or interdigitating leaf pavement cells [26]. Yet, the most conspicuous wall patterns are arguably those emerging during SCW deposition. SCW thickenings exist extensively in the plant kingdom, in red algae as well as bryophytes, lycophytes, ferns, gymnosperms and angiosperms [27–29]. Various SCW patterns may be found around a range of different cell types, including fibres, anthers, seed coats and trichomes [30,31]. However, the most well-studied SCW patterns are those occurring in xylem TEs, which include annular, helical, scalariform, reticulate and pitted wall patterns (figure 1). In this part, we briefly outline the organization of the xylem tissue and the way SCW patterning support xylem function.

TEs carry out their main function when they are dead, and thus void of protoplasm. The SCW patterns allow for a highly interconnected TE system that efficiently transports water and minerals throughout a plant and that, together with fibres, provide for plant stature. Water transport is facilitated by several factors. Firstly, roots contain a higher concentration of solutes compared to the surrounding soil, which osmotically draws water into the root hairs [32]. The water then moves up the stem, against gravity, thanks to the effects of positive ‘root pressure’ and capillarity [33]. However, water pressure in the roots and capillarity alone only partially justify the movement of water up the stem. In fact, the main driver of water transport through the vascular system is evaporation from the surface of leaves (transpiration) via the stomata, which open for gas exchange during photosynthesis [34]. Considering the remarkable cohesive properties that water molecules have, it is unsurprising that evaporation from the stomata generates a negative pressure that induces suction of water from the stems to the leaves. The lignin-rich SCWs allow for the TEs to resist the negative pressures that are generated from these different factors [35].

Vessels and tracheids show significant differences in structure, size and water conduction efficiency. Vessels, almost exclusively found in angiosperms, typically have larger diameters and lengths. They have a cylindrical shape with slightly diagonal cell ends, through which they are connected to each other longitudinally via perforation plates that facilitate water passage [36]. Vessels are syncytia, meaning that they are formed by many piled-up single cells, called vessel elements. By contrast, tracheids are elongated cells with tapered ends and, in some cases, a polygonal cross-section [37]. They do not contain typical perforation plates that would connect them to other tracheids, but they are connected laterally. These cells are narrower, shorter and relatively less developed than vessels, with a thicker SCW. Although prominent in gymnosperms, they are present in all vascular plants [38]. The imperforate TEs in angiosperms xylem can be divided into three types: tracheids, fibre-tracheids and libriform fibres. These three cell types may be distinguished by the number and shapes of pits and lignin content [39].

During plant growth and development, the xylem changes to fit the requirements of the surrounding tissues. For instance, during primary growth, the primary xylem is formed (inwards) from the procambium—a meristematic tissue that drives vasculature formation—along with primary phloem (outwards). At this stage, a protoxylem, typically consisting of small SCW producing cells, is generated within the primary xylem, followed by metaxylem, which has larger cells [40]. Metaxylem (and metaphloem) cells are developed from fascicular cambium (a cambium differentiated from the pro-cambium and located in-between xylem and phloem). Functionally, protoxylem cells can continue to extend, whereas this does not occur for metaxylem cells. One reason for this is a difference in SCW patterns around the two cell types: annular or helical SCWs in protoxylem and reticulate or pitted in metaxylem [40]. While annular and helical cell wall thickenings allow for further cell elongation, reticulate and pitted thickenings provide greater strength to support larger volumes of water, transported over longer distances [22]. Once secondary growth is initiated, secondary xylem (or wood) can develop from a secondary meristem called vascular cambium. While vascular cambia can be found in gymnosperms and dicotyledonous angiosperms, they are not present in monocotyledons [41]. Therefore, monocots, such as *Bracypodium distachyon,* develop scattered vascular bundles throughout the stems [42,43]. By contrast, the organization of bundles in most eudicots is in a ring-like pattern, with bundles being linked by thick interfascicular fibres [43–45]. Gymnosperms, on the other hand, lack fibres and xylem vessel elements, although the bundles can also be found organized in a ring-like form [39,44]. In *Cycas*, for example, the central pith is surrounded by the vascular bundles. In seedlings, the vascular cylinder is mesarch (metaxylem develops on both sides of protoxylem), while in adult plants it becomes endarch (metaxylem develops centrifugally). *Cycas* bundles are collateral (i.e. the xylem grows inwards and the phloem outwards) [46].

Although annular and helical SCW patterns are generally associated with protoxylem, there are quite a few variations in terms of their arrangements and localization. Studies on *Arundo donax* and *Phyllostachys aurea* show that protoxylem vessels can have annular thickenings with large diameters as a single bundle [47]. Helical patterns can also be found in tracheids, such as those in *Pinus densiflora* [48] and fibres, e.g. ground tissue fibre in *Loropetalum* [49]. Annular deposition of thick SCWs in mature genicular cells in *Calliarthron cheilosporioide,* provides flexibility and makes this red alga resistant to bending stress and breakage under waving forces [28,50]. Finally, alternate bordered pits and helical thickening are commonly found in the vessels of woody climber *Clematis vitalba*, to provide lianas with extraordinary stem flexibility and long-distance water conduction capacity [51,52]. When protoxylem progresses into metaxylem, helical pattern can evolve to form irregular nets called reticula.

Reticulate patterns can be found in both vessels and tracheids in angiosperms [17]. Pitted patterns provide connections between metaxylem vessel elements and other differentiated cells, such as adjacent elements, ray parenchyma cells, axial parenchyma cells, tracheids and fibres [36]. Intervessel pit shape and arrangements vary, forming scalariform, opposite or alternate pit patterns and differ in different cells and plant species. The two main typologies of pits are simple and bordered, where the borders are composed of concentric microfibrils around the pit [53]. Alternate bordered pits tend to have circular, oval and polygonal outlines. In one anatomical observation of roots of *Zingiberaceae* species, wider vessel walls consist of scalariform perforation plates, while the narrower ones have rounded rectangular or oval elliptical pits. In addition, in mature vessels, the perforation plates completely lacked pits [54,55]. Combination of different pit patterns occurs in some hardwood species [56–59]. For example, *Ceiba speciosa* has alternate and polygonal intervessel pits, while it has bordered vessel-ray pits and round pits between vessel-parenchyma cells [60]. Pitted patterns among the three cell types of imperforate TEs are different. Tracheids, for instance, have numerous circular bordered pits that contribute with water and minerals transport. Fibre-tracheids, such as the ones in *Acer rubrum* have fewer and smaller-bordered and silt-like pits. Finally, libriform fibres—like the ones in *Vitis* spp.—have reduced number of simple pits with silt-like aperture [39]. Imperforate TEs in angiosperms have four types of perforation plates, namely simple, scalariform, reticulate and foraminate. In some cases, multiple perforation plates are observed [61].

While the above relates to xylem SCW patterning, other tissues contain SCW patterns that differ from those listed. One prominent example is the donut-shaped and pectin-rich SCWs that occur in seed coat epidermal cells in, for example, *Arabidopsis thaliana* (thale cress; hereafter 'arabidopsis') [62]. This specialized SCW can burst the overlaying primary wall upon contact with water to create the typical mucilage halo surrounding many seeds. Another case in arabidopsis is that of lignified SCWs in the endocarp. Lignified SCW patterns, associated with the valve margins of the endocarp, are key to silique rupture and thus seed dispersal [63].

Lastly, a unique pattern is the striated SCW thickening discovered in the anther endothecium cells. Alternation in these patterns affects anther dehiscence. In arabidopsis, absence of cell wall thickenings prevents anthers from opening and releasing pollen, while, in cotton, abnormal thickenings (i.e. transversal SCW) in endothecium hindered anther dehiscence [64–66]. Observation of anthers in rice revealed that the U-shaped thick cell wall in the endothecium helps maintain the pressure needed to split and overflow the mature pollen [67]. To sum up, there is a plethora of SCW patterns associated with different plant tissues and species. These patterns can change during development but also in response to environmental conditions to support a variety of functions. The formation of these patterns is not well explored and thus, in most cases, the regulatory framework that underpin the patterns remain largely obscure.

## 3. Systems to study secondary cell wall patterns

**Figure 2.**
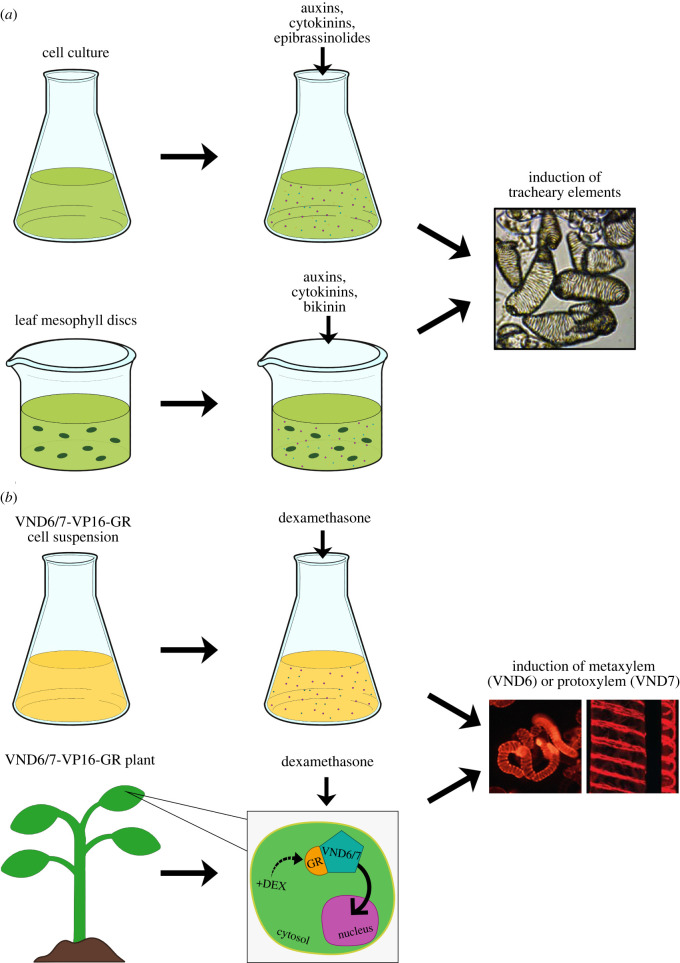
Systems to study cell wall patterns. (*a*) Hormones and hormone-related molecules can induce xylem transdifferentiation. (*b*) Adding dexamethasone to the VND6/7-glucocorticoid receptor (GR) systems induces metaxylem (VND6) and protoxylem (VND7; image from *A*. *thaliana* hypocotyl cell) formation.

This section addresses how SCW patterns can be induced, for research purposes, in cells and tissues that do not ordinarily make them. We have compiled an overview of these systems to accompany the text below (figure 2).

### 3.1. Induction of xylem transdifferentiation by hormones and related molecules

Since as early as 1855 [68], *Zinnia elegans* (hereafter 'zinnia') cells cultures have been used to study *in vitro* transdifferentiation of xylem cells, which is accomplished by supplying different types of phytohormones, such as auxin and cytokinin, to promote TE formation [69–72]. This system allowed researchers to investigate the function of genes involved in TE differentiation, highlighting, for instance, the cortical microtubules bundling function of the microtubule-associated protein MAP65-1 [73–77]. As the arabidopsis genome became available, efforts to use this species to study transdifferentiation intensified, leading to the identification of several regulatory genes, including the so-called master regulators of xylem TE differentiation, or VASCULAR-RELATED NAC-DOMAIN6/7 (VND6/7; [78]). While different hormone cocktails have been used to induce transdifferentiation, the optimization of the best-inducing combination has proven quite tedious. For example, a certain mixture of NAA, BAP (an auxin and a cytokinin, respectively) and epibrassinolides reached an induction efficiency of TEs of about 40% in arabidopsis cell cultures [79–81]. Brassinosteroids (BR) are a key class of hormones for TE differentiation. For instance, brassinolide causes an upregulation of VND6 but represses both VND6 and VND7 when combined with cytokinin. The latter cannot induce the differentiation of xylem fibres when lacking indoleacetic acid and gibberellic acid [82,83]. In zinnia cell cultures, the addition of uniconazole (an inhibitor of BR biosynthesis) combined with auxin and cytokinin, prevented TE differentiation in mesophyll cells, while exogenously applied BR counteracted the inhibition [84]. Additionally, in cultured zinnia cells, ethylene is an essential plant hormone for *in vitro* TE differentiation, during which its biosynthesis was found to be boosted. Further, inhibition of ethylene biosynthesis via application of pharmacological inhibitors of the enzymes that produce ethylene precursors hindered TE differentiation in zinnia [85]. These data indicate that cocktails of different hormones drastically influence TE differentiation.

Apart from the specific hormones, hormone-related molecules and proteins have also found application in SCW induction. An example is represented by the histidine-containing phosphotransfer factor 4 (AHP4), a protein involved in cytokinin signalling propagation that negatively regulates SCW thickenings [86]. Similarly, the small signalling dodecapeptide Tracheary element Differentiation Inhibitory Factor (TDIF), through its receptor protein, TDIF RECEPTOR (TDR), interacts with and activates glycogen synthase kinase 3 proteins (GSK3 s), which suppress xylem differentiation [87]. GSK3 s include BRASSINOSTEROID INSENSITIVE2 (BIN2), a central negative regulator of the BR pathway, unraveling a potential link between the TDIF and the BR signalling pathways. In this context, leaf mesophyll transdifferentiation does not normally occur in the presence of auxin and cytokinin alone; however, application of the GSK3 s inhibitor, bikinin, along with the two hormones, effectively induces differentiation into procambial cells and, later, into TEs in arabidopsis. This highlights the importance of the BR signalling pathway and a direct role for TDIF in controlling xylem differentiation [88,89]. The peptide sequence of TDIF/CLE41/44 is conserved among most herbaceous and/or woody/shrubby/perennial dicots, with only a single amino acid difference, but is not found in the majority of monocots, except in *Phoenix dactylifera* (date palm) [90]. Similar to other peptide-receptor pairs, the binding affinity is amino acid sequence-dependent [91], and changes to the peptide sequences may thus substantially change the phenotypic outcomes [90,92,93].

Other peptides that impact TE development, include the endogenous sulfated pentapeptide phytosulfokine, which stimulates TE differentiation in zinnia cell cultures, although proper induction requires once again the addition of auxin and cytokinin [94]. In addition, xylogen, an arabinogalactan-related protein, was initially inferred to be associated with xylem development from observations in zinnia cell cultures. As the name suggests, xylogen is involved in the xylem transdifferentiation process and mutation in this protein results in partial loss of xylem, in arabidopsis [95,96]. The use of a range of hormone cocktails and associated signalling pathways may thus be exploited to induce xylem transdifferentiation in a range of tissues and species [97]. This approach nicely complements that of different genetic approaches to induce SCW synthesis.

While the tissue cultures are remarkable tools for studying the formation of xylem elements *in vitro*, there are several drawbacks in mimicking *in planta* processes. Indeed, the systems are dependent on a cell culture environment with exogenous growth regulators that can be easily disrupted by the addition of dyes just before the onset of differentiation, leading to formation of truncated, sinuous, or smeared SCWs [98]. In addition, the regulation of cell size or shape seems to be an important factor for SCW patterning. Studies with the zinnia mesophyll cell system showed that cells with increased cell width, and in higher pH conditions, were more likely to form metaxylem-like TEs [72,99]. This pH-dependent cell-shape-related SCW patterning is controlled by the remodelling of the cortical microtubule cytoskeleton [100] (see also below). Studies with mutagenized arabidopsis seedlings found that several cell expansion-related mutants showed defects in SCW lignification. For example, mutants of ECTOPIC LIGNIFICATION1 (ELI1; a catalytic subunit of cellulose synthase), LION'S TAIL (LIT), WOODEN LEG (WOL), RADIAL SWELLING1 (RSW1, another catalytic subunit of cellulose synthase), KORRIGAN1 (KOR1, a β-1,4 endoglucanase) and DE-ETIOLATED-3 (DET-3), stall cell growth and cause ectopic lignification in xylem cells [101–106]. However, the relation between cell size and shape, and SCW patterning is still unclear. Moreover, in a study of the phenotype of GAPPED XYLEM (GPX) mutant in arabidopsis, perforation plates appeared in gaps between xylem elements [107]. However, in the zinnia mesophyll cell system, mesophyll cells are induced to differentiate into single xylem elements without formation of perforation plates [108], which makes it difficult to study the function of perforation plates in this system. Hence, while these culture systems are useful to deduce molecular components that impact xylem formation, there are certainly processes where these systems are not suitable.

### 3.2. Inducible VND systems to study secondary cell wall development

The identification of VND6 and VND7, and the finding that they can induce SCW synthesis [78], has provided important tools to understand xylem development. The VND proteins belong to a much a larger family of NAC-DOMAIN transcription factors which are conserved among a wide range of plant species [109,110]. These include the master regulators of xylary fibres NAC SECONDARY WALL THICKENING PROMOTING FACTOR1 (NST1), NST2 and NST3/SECONDARY WALL RELATED NAC DOMAIN1 (SND1) [111–114]. Other members include SOMBRERO (SMB) which although only expressed in root caps and root meristems, can induce SCW formation when expressed ectopically [115]. These data suggest that this family of transcription factors, collectively called VNS (VND, NST/SND, SMB), have a conserved ability to activate SCW synthesis and evolved from a common ancestral gene that is thought to control cell wall modifications during differentiation of ancestral water-conducting cells [109].

Of the seven VNDs in arabidopsis, VND6 and VND7 are key regulators in vessel element development. Dominant repression of *VND6* and *VND7* inhibits formation of metaxylem and protoxylem, respectively [78]. Additionally, overexpression of *VND7* or *VND6* is sufficient to drive ectopic differentiation of many cell types into protoxylem-like and metaxylem-like vessel elements, respectively [78]. These results provided the basis for the generation of transgenic arabidopsis lines that constitutively express VND6/7 fused with the VP16 activation domain and a glucocorticoid receptor domain (VND6/7-GR). In the absence of the glucocorticoid, the VND6/7-GR proteins are retained in the cytosol and thus not active. However, upon addition of dexamethasone (a glucocorticoid not present in plants), the VND6/7-GR proteins can translocate from the cytosol to the nucleus, thus activating downstream genes related to SCW production. Indeed, within a few hours, vessel-like cells appear with SCWs arranged in either annular/helical patterns in VND7-GR lines or pitted/reticulate patterns in VND6-GR lines [116]. Notably, and in contrast to phytohormone-induced systems [71,81,117], these systems specifically produces only protoxylem and metaxylem vessel-like elements, respectively [78]. The synchronized differentiation of cells into protoxylem/metaxylem vessels has allowed researchers to analyze the changes in the transcriptome [116,118], metabolome [118,119] and cellular dynamics of SCW formation [118,120–126].

Furthermore, variants of the inducible VND6/7 system have been developed to improve the reliability and applications of the systems. For example, Oda *et al*. established a cell culture system in which oestrogen-inducible VND6 promotes metaxylem vessel differentiation in arabidopsis suspension cells [127]. Upon application of oestrogen and brassinosteroid, over 80% of cells synchronously differentiate into metaxylem vessel cells within 32 h [127,128]. Together with complementary transient transformation techniques, this system enabled high-throughput imaging of differentiating metaxylem cells. Using this system, many downstream genes of *VND6* were identified and, through subsequent analyses, placed in context of metaxylem differentiation [127,129]. As a result, several key genes that regulate formation of SCW pits were identified [127,130–136] (see also below).

The VNDs have been used in several eudicots, including arabidopsis, but also more recently in other plant species confirming their conserved functions as master regulators of xylem vessel development. In the model tree species *Poplar trichocarpa* (poplar), VND genes are expressed in developing xylem [137,138], and heterologous overexpression of arabidopsis VNDs and poplar VNDs in either species resulted in ectopic xylem vessel differentiation [137,138]. Similar results were also found for VND homologues in model monocots such as a *Oryza sativa, Zea mays and Brachypodium distachyon* [139–141]. This conservation in function also extends to other species outside eudicots with other types of water-conducting cells. In the gymnosperm *Pinus taeda,* VND homologues VNS1-5 are master regulators of xylem tracheid development [142]. The VNSs may also drive ectopic differentiation of xylem vessels when expressed in either tobacco or arabidopsis [142]. Interestingly, this conservation in function also extends to the hydroids of the moss *Physcomitrella patens* [110]. Together these results highlight the conserved function of the VNS through the evolution of land plants and include conserved downstream targets. While the VND-inducible systems can be used across several different types of plant cells (e.g. suspension cells, transient infiltration systems and stable transgenic plant lines), there are of course drawbacks, for example, related to the lack of naturally surrounding xylem cell environments. Nevertheless, both the hormone-inducible suspension culture systems and the various VND-inducible systems offer powerful tools to study the molecular and evolutionary process of xylem TE differentiation across many different plant species.

## 4. Drivers of xylem cell wall patterns

**Figure 3.**
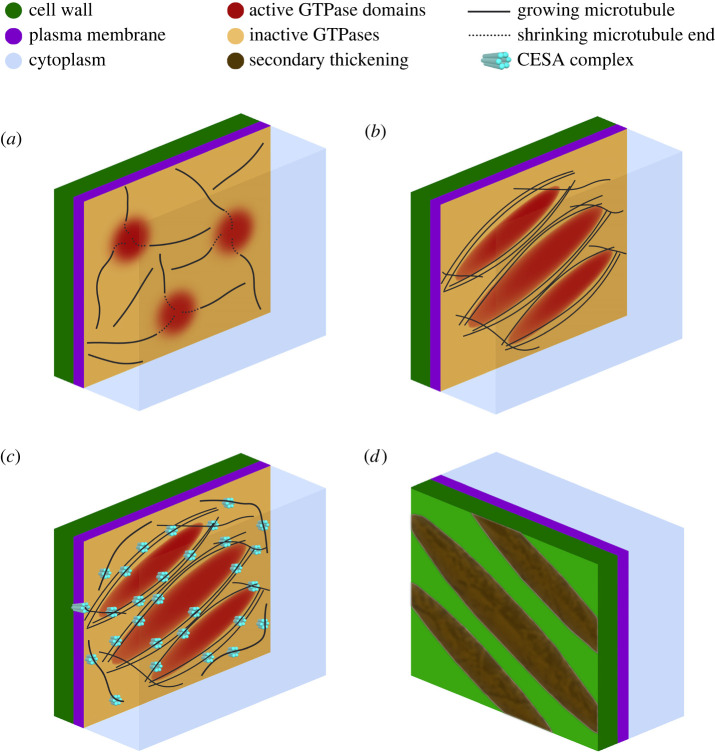
Drivers of xylem cell wall patterns. (*a*) ROP domains formation in the presence of a primary wall-like microtubule cytoskeleton. Microtubule ends are targeted in the active ROP domains, while microtubules remain unperturbed outside the ROP domains. (*b*) Active ROP domains are shaped in a microtubule-dependent manner. (*c*) SCW synthesis machinery (CESA complexes) is recruited. (*d*) Patterned deposition of SCW material.

The above xylem induction systems have been crucial to our understanding of SCW patterning. In essence, patterned SCWs require three cellular entities to work in concert: the plasma membrane, the microtubule cytoskeleton and the cell wall synthesis machinery [143]. Therefore, the question of what drives the formation of cell wall patterns during xylem development has to take into account pattern formation processes taking place for each of the three constituents and then how they link to each other (figure 3).

### 4.1. Cellulose synthesis during wall patterning

Cellulose is a central component of both primary and SCWs [144]. The glucan polymers that make up cellulose are synthesized at the plasma membrane by cellulose synthase (CESA) proteins [145]. In many plants, the CESAs form hexameric rosette complexes (CSC) that move during synthesis. The movement is probably fuelled by the catalytic activity of the CESAs; the stiff cellulose fibres are immobilized by entanglement in the wall structure and further synthesis therefore thrusts the CSC forward in the membrane [125,143,145]. The direction of the movement is controlled by cortical microtubules through the CESA connected protein CELLULOSE SYNTHASE INTERACTING1 (CSI1) and COMPANION OF CELLULOSE SYNTHASE (CC) proteins [121,146–150].

While CSI1 appears to be a constant anchor to CSCs during both primary and SCW formation, primary wall CSCs are exchanged for SCW CSCs during the wall transition [126]. Notably, the primary wall CSC consists of three CESAs, CESA1, CESA3 and one of the CESA6-related proteins in arabidopsis; however, the SCW CSC holds CESA4, CESA7 and CESA8 [151–153]. Live cell imaging using the VND7-GR inducible protoxylem system, with members of both the primary and SCW CESAs fluorescently tagged (tdTomato-CESA6 and YFP-CESA7, respectively), revealed that this happens in a step-wise fashion, where the primary wall CSCs are slowly turned over in the vacuole, while SCW CSCs are expressed and delivered to the plasma membrane [126]. Yet, for a brief period, primary and SCW CESAs coexist at the plasma membrane. However, detailed analysis of CESA velocities showed that SCW CSCs moved significantly faster than primary CSCs, demonstrating that the bulk of their activity is as separate complexes. The different speed of the two CSCs, and the brief overlap of them in a single cell, indicate that there is little room for ‘mixed’ primary and SCW CSCs [154]. Nevertheless, these analyses cannot rule out that possibility.

SCW CSCs seem to massively populate the microtubule bands and produce cellulose within a short time window of only a few hours, maybe driven by the need to rapidly produce SCWs [126,155]. It appears that this process is accompanied by targeted exocytosis via the exocyst complex [124] (see also below) and delivery of other SCW polymers such as xylan and lignin [24,156]. It is, however, not clear how the SCW CSCs can synthesize cellulose at a faster rate than primary wall complexes. One obvious candidate for such changes could lie in divergence of post-translational modifications, such as changes to phosphorylation sites of the CESAs, which may change the speed and tracking behaviour of the CSCs [157,158].

### 4.2. The microtubule cytoskeleton

Given the central role of microtubules in steering the synthesis of cellulose, it appears clear that the microtubules need to undergo substantial re-organization to support SCW patterns. Primary wall cellulose is supported by a diffuse microtubule array that direct anisotropic cellulose deposition and consequently changes in cell growth patterns and shape [159]. During SCW synthesis, the microtubules dramatically change their organization to support corresponding cell wall patterns [117,122]. Consequently, the microtubule array changes into a helicoidal/banded array during protoxylem formation and similarly into a reticulate patterning during metaxylem formation [121,122,128,131,132,134,160,161]. These microtubule configurations then direct SCW deposition. Indeed, perturbation of microtubules, by treatment with the microtubule-depolymerizing drug oryzalin, results in the improper arrangement of the SCW CSCs in cells at the early stages of VND7-GR induction [121,125]. Interestingly, oryzalin treatment at later stages of VND7-GR induction, when microtubule banding has already begun to occur, results in only partially perturbed SCW patterning [121]. This observation highlights that CESA trajectories during cellulose synthesis can not only be guided by microtubules, but also by the tracks of cellulose microfibrils already present in the wall [121,162]. Still, without the initial guidance provided by the microtubule re-organization, bundled organized tracks of cellulose do not form, thus indicating the importance of microtubules in outlining tracks of cellulose production during SCW formation.

In addition to CSC trajectories, microtubules also play an important role in guiding CESA delivery at the plasma membrane. Similar to primary wall CESAs, SCW CESAs are preferentially delivered to the plasma membrane in close vicinity of microtubules [125,126,163]. By contrast, in the absence of microtubules, CESA delivery occurs relatively evenly throughout the plasma membrane [125]. This targeted delivery of CESAs to microtubule-lined domains is believed to be directed in part by exocyst complexes. The exocyst is a large multimeric protein complex involved in the tethering of secretory vesicles to the plasma membrane and preliminary data has indicated that it may be recruited to cortical microtubules [124,133]. Indeed components of the exocyst complex co-localize with microtubule bundles in VND7-GR induced cells and mutations to exocyst complex subunits results in perturbed SCWs [124,164]. These aberrant walls are in part explained by the mislocalization of CESAs, which indicates that the exocyst is required for proper CESA trafficking [124]. Additionally, it has been speculated that local changes in the membrane content might also contribute to targeted delivery [25,121]. Plasma membrane deformations may also influence CESA delivery preferences. As the CSCs produce cellulose, mechanical tension is built up within the synthesized cellulose fibres, which on the one hand drives the propulsion of the CSCs within the membrane, but probably also causes depression or at the very least considerable tension of the plasma membrane around the CSC [165]. This will probably result in a change in the membrane morphology and perhaps even forming a barrier for certain organelles inside the cells, which could contribute to uneven delivery events. Nevertheless, lack of coordinated delivery patterns and subsequent guidance of the CESAs by microtubules result in aberrant and disorganized tracks of cellulose microfibrils and thus loss of SCW patterns [121,125].

Apart from cellulose, the microtubules appear to also guide the delivery of other cell wall components. Indeed, microtubule disruption resulted in perturbations of xylan deposition in the SCW of xylem vessels in angiosperms [156]. Like the majority of cell wall polysaccharides, hemicellulose biosynthesis occurs in the Golgi and is trafficked via the trans-Golgi network (TGN) to the plasma membrane [24,120]. The xylan-containing secretory vesicles are targeted to sites of SCW synthesis, apparently via microtubules, again by an unknown mechanism [156]. One aspect of such mechanism may be via a common delivery system, as the altered patterns of xylan deposition closely followed the patterns of cellulose microfibrils in microtubule-depolymerized cells [156]. This may indicate that secreted polysaccharides and CESA enzymes may traffic together to the plasma membrane in the same vesicles; however, this circumstantial observation needs to be further investigated. Interestingly, a recent study nicely show that xylan occurs as nano-domains at the pit borders to control pit size and shape during metaxylem formation [166], perhaps by directly interacting with cellulose fibres [167] supporting the microtubule and CSC patterns. As indicated above, one of the key complexes that could be involved in targeting vesicles containing hemicellulose and secreted glycosylated proteins is the exocyst complex. However, the same mutations to exocyst components that results in partially mislocalized CESAs do not perturb the banding pattern of laccases, a set of key enzymes involved in the polymerization of lignin [124]. Thus indicating that laccases and potentially other secreted glycoproteins may be targeted to microtubule-lined domains via some other unknown pathway.

### 4.3. Patterning the plasma membrane

The different types of wall patterns are probably premediated by those forming in, or at, the plasma membrane. Such changes are thought to occur before changes in the microtubule networks become visible and the cell wall machinery is expressed and delivered to the plasma membrane [121,122,126]. Small GTPase proteins have proven critical in *de novo* formation of membrane patterns in many different organisms [130,168–170]. In plants, such a role is provided by 11 highly conserved Rho of plants (ROPs). Like all small GTPases, ROPs possess two states: a GTP-bound active state and a GDP-bound inactive state. It is the active state that facilitates transient interactions with effector and regulatory proteins that can induce periodic activation cycles of signalling cascades [171,172]. Inactive ROPs are activated by guanine nucleotide exchange factors (GEFs), which aid in replacing GDP with GTP. Several studies showed that small GTPases and their associated GEFs can directly interact, leading to quick re-activation [130,131]. In turn, ROPs are inactivated by GTP hydrolysis, which is accelerated by GTP-activating proteins (GAPs). Active ROPs are membrane-bound due to post-translational modifications that render the C-terminal hydrophobic either via prenylation (ROP1 to ROP8 or type-I ROPs) or S-acetylation (ROP9 to ROP11 or type-II ROPs) [171]. Prenylated ROPs can be sequestered from the plasma membrane by cytoplasmic guanine dissociation inhibitors (GDIs) whereas S-acetylated ROPs can remain at the plasma membrane even when inactive [171,173]. The diffusion constant of proteins in the plasma membrane is approximately three orders of magnitude slower than in the cytoplasm, and together with the two-state reaction cycle of ROPs, provides the ideal conditions for a classic patterning process called Turing-like reaction–diffusion (RD) mechanism [168,174].

A Turing-like RD system consists of two diffusible substances that can interact (or ‘react’) with each other and has the potential to autonomously produce spatial patterns [168]. In absence of interaction (diffusion alone), such systems can still produce complex patterns that would, however, depend on external conditions such as local gradients or pre-patterns of a ‘morphogen’. The addition of the interaction renders this system to become autonomous (i.e. producing *de novo* patterns independent of any pre-pattern) [168]. Many theoretical studies have used such models to explain spatial patterns in organisms as diverse as seashells [175], fish skin and animal furs [176,177], the slime mold *Dictyostelium discoideum* [178] and the model plant arabidopsis [130,179,180].

## 5. Patterning frameworks for proto- and metaxylem

The general framework for SCW patterning is outlined in the above section. Here, we will outline how specific components contribute to proto- and metaxylem SCW patterning.

### 5.1. The protoxylem SCW patterns

As mentioned in the introductory section, the protoxylem forms when the surrounding tissue still elongates and consists of a periodic pattern of SCW bands or coils. Such patterning allows for the continued elongation of the vessel element even after the cell has undergone programmed cell death to accommodate the surrounding expanding tissues. Expression and proteomic experiments suggest that specific ROPs, GAPs and GEFs are expressed during protoxylem patterning [78,116,118,129,181]. ROP7 was identified as a xylem-specific GTPase; however, its role during protoxylem development has not been yet investigated [182]. Shedding light on the active ROPs (and associated control GEFs and GAPs) and their regulation of protoxylem formation is thus of great importance to the advancement of this field. Theoretical studies regarding how Turing-like RD processes may operate in protoxylem revealed that diffusion anisotropy, as produced by microtubule-based diffusion restriction, is critical to this system. Under such conditions, a Turing-like RD mechanism favours banded patterns whose orientation is defined by the overall orientation of the diffusion anisotropy [160]. Interestingly, this outcome is independent of whether these systems would form spot, stripe or gap patterns in absence of the diffusion anisotropy. These findings may explain why the protoxylem pattern seems inert against mutations in ROP11, ROPGAP3/4, ROPGEF4/7, because another ROP-GAP-GEF combination may in principle take over the role of a patterning ‘system’ even if its biochemical properties would normally drive different patters [131] (see below).

Given these implications, it seems obvious that microtubules have to re-orient transversely to support band patterning in protoxylem. In fact, microtubules transition from unordered into banded arrays with homogeneous microtubule orientations [121,122,125,126]. From these studies, it became clear that the microtubule re-orientation into banded arrays occurs simultaneously in each cell which indicates that a cell-wide pre-pattern is steering the microtubules [122]. Furthermore, the dynamics of microtubules is tightly controlled in space (between gaps and bands) and time (microtubule response occurs 24 h after induction but lasts for only 3 to 4 h to accomplish a pattern), which further indicates that regulatory proteins (such as GEFs and GAPs) fine-tune this process [122]. Proteomic analysis has uncovered several microtubule-associated proteins, such as MAP70-1 and MAP70-5, which support the arrangement of microtubules within bands [79,81,183]. The nucleation of microtubules needs to be locally controlled as simulations of dynamic microtubules revealed that the strongest microtubule band would otherwise sequester all free nucleation complexes leading to over-amplification of a single band [122]. How such local control is achieved is another open question, but it may be due to spatial restrictions in the diffusion of the nucleation complexes.

From the above studies, it is established that cortical microtubules template SCW cellulose deposition [121,125,126,184]. Along with cortical microtubules responding to the underlying pre-pattern comes the re-orientation of the primary CSC machinery. However, this process seems partly independent of microtubules as plants lacking the CSI protein still produce banded cell walls [121]. It should, however, be noted that not all CESA trajectories deviate from the microtubules in most of the *csi1* mutants. There could, therefore, be additional components that contribute to the alignment, such as the more recently discovered CC proteins [147,149]. Nevertheless, there is a clear possibility that interactions between the CESAs/cellulose and other wall components may support the transition and tracking patterns. Indeed, primary wall CESAs are guided not exclusively by microtubules but also possess a microfibril-based guidance system [162].

### 5.2. The metaxylem SCW patterns

By contrast to protoxylem, metaxylem form when cell expansion has ceased, thus form less flexible but more resilient SCW patterns, such as pitted or reticulate. Such structures allow for increased vessel diameter and wall strength, which allows for greater water flow. Several studies using histochemistry revealed that cortical microtubules are present beneath SCWs but absent inside the pits [185–190]. Although some researchers speculated that the patterns of pitted or reticulated walls are gradually developed from simple patterns, such as helical or annular patterns [117,191], the underlying processes long remained unclear.

**Figure 4.**
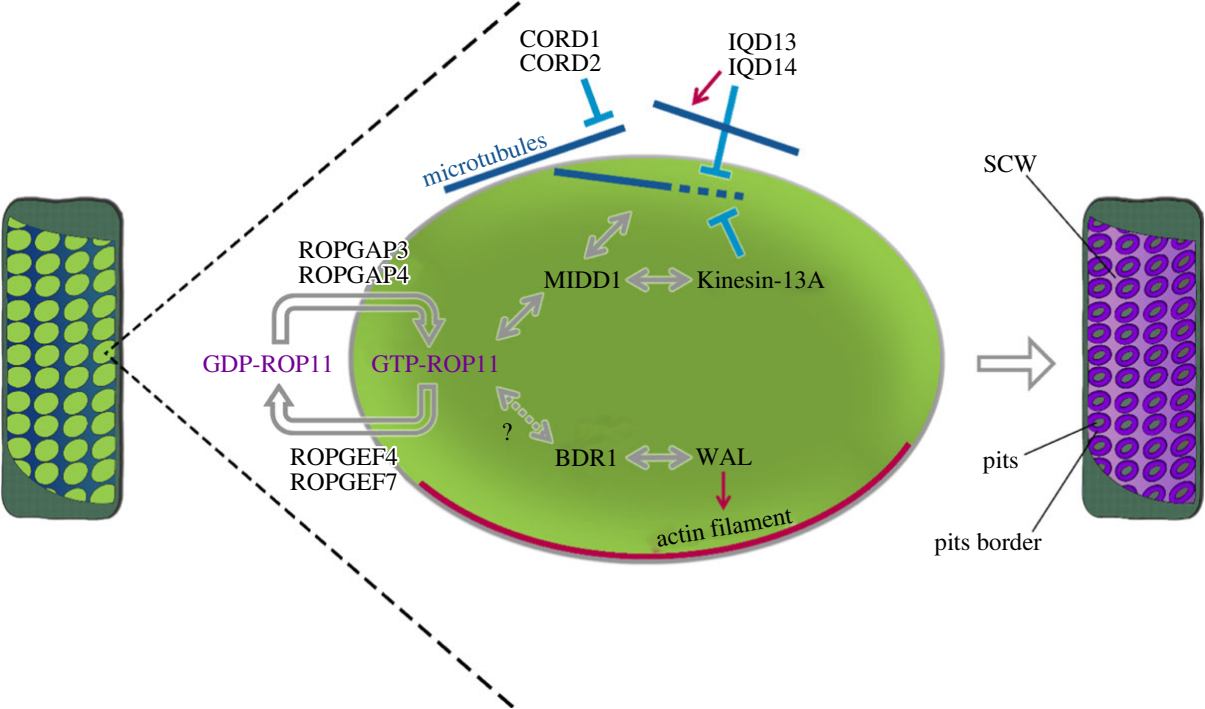
A schematic model of regulation of secondary cell wall development in metaxylem vessels. Light green domains (ovals) indicate plasma membrane domains marked with activated ROP11. Double arrowheads indicate interactions. Red arrows indicate promotion of bundling and/or polymerization. Light blue bars indicate elimination of microtubules or activated ROPs.

By using the VND6-inducible arabidopsis cell culture, Oda *et al*. revealed that cortical microtubules gradually disappear from developing pits [127]. MICROTUBULE DEPLETION DOMAIN 1 (MIDD1), a member of Interactor of Constitutively Active ROP/ROP-interactive Partner (ICR/RIP) family, is required for this process (figure 4). MIDD1 is specifically recruited at the plasma membrane domains by activated ROP11 and accumulates at the plus ends of cortical microtubules growing into these domains [127,131]. MIDD1 interacts with Kinesin-13A [132,192], a class-13 member of the kinesin superfamily. Animal members of kinesin-13 are known to processively depolymerize microtubules from both ends [193,194]. Although Kinesin-13A alone does neither bind to nor depolymerize cortical microtubules *in vivo*, interaction with MIDD1 causes the recruitment of kinesin-13A to microtubule ends in pit domains, and therefore their efficient depolymerization [132]. Thus, MIDD1 links changes in microtubule dynamics to ROP11-activated plasma membrane domains. However, it remains unknown whether ROP members other than ROP11 are involved in setting up such a ‘prepattern’ at the plasma membrane.

### 5.3. ROP signalling regulates pit pattern

Members of GEF and GAP families that are unique to plants, ROPGEF and ROPGAP, respectively, regulate ROP11 to facilitate pit patterns of metaxylem vessels (figure 4). In VND6-induced suspension cells, ROPGEF4 and ROPGAP3 are localized at the plasma membrane in the SCW pits where ROP11 is exclusively activated. When ROPGEF4 and ROPGAP3 were co-expressed together with ROP11 in non-xylem cells, ROPGEF4 formed numerous patches of dotted patterns at the plasma membrane with locally activated ROP11 within these patches [130,131]. An RD model descripting the ROP reaction cycle, implemented with positive feedback loops, indicated that the ROP reaction cycle can cell-autonomously produce domains where active ROP is enriched [130]. These findings indicate that the ROP activation cycle spontaneously generates dotted patterns of ROP11 at the plasma membrane and that these then drive the pitted SCW patterns. In fact, altering the expression levels of ROPGEFs and/or ROPGAPs influences the density and size of SCW pits [130], which suggests that these three components are sufficient for the patterning system to be functional and tunable.

### 5.4. IQD13 and CORD1 regulate pit shape

As described above, pits of various sizes and shapes are found in different angiosperms and in response to environmental conditions [195]. The pits in arabidopsis metaxylem are majorly of oval shape, indicating that their shape control underlies strict regulation. By using VND6-iducible cell suspensions, two homologous proteins from the IQ67 Domain (IQD) family, IQD13 and IQD14, were found to regulate the oval shape of SCW pits of metaxylem vessels. IQD13 associates with the plasma membrane and increase the density of cortical microtubules [136]. Indeed, IQD13 can associate with microtubules to form molecular ‘fences’, which confine the diffusion of active ROPs in the membrane. Thus, IQD13 likely regulates the pit shape by controlling the permeability of the microtubule fences, which in turn restrict the shape of the active ROP-labelled domains [136].

Furthermore, CORD1 (CORTICAL MICROTUBULE DISORDERING1) and its paralogue CORD2 were identified as regulators of pit shape [134]. CORD1 is localized to cortical microtubules and randomizes the array by promoting detachment of microtubules from the plasma membrane. Due to these activities, CORD1 probably prevents cortical microtubules from acting as molecular fences, almost counteracting IQD13, and thereby influences shape of ROP domains and subsequent pit shape [134]. The balance of IQD13 and CORD1 levels may thus determine the shape of ROP domains from narrow to enlarged, thereby determining the oval shape of SCW pits (figure 4).

### 5.5. BDR1 and WAL direct pit border formation

In differentiated metaxylem vessels, SCWs massively accumulate at pit boundaries to form ‘bordered pit structures', creating the window that serves as the lateral passage for xylem sap. Several studies imply that actin microfilaments are involved in the formation of this structure [196]. In cultured zinnia mesophyll cells, actin microfilaments accumulate at pit regions and influence the SCW pattern [197,198]. In the secondary xylem of tree species, actin microfilaments are present along the pit boundaries, forming actin rings [196,199]. In arabidopsis, actin rings are also present at the pit border of metaxylem vessels [135]. Treatment with an actin-depolymerizing agent results in the loss of SCWs at pit boundaries, indicating that the actin ring is essential for the bordered pit structures [135]. However, molecules involved in these actin rings have long been unknown.

Again, using VND6-inducible suspension cells, two proteins, named WAL (WALLIN) and BDR1 (BOUNDARY OF ROP DOMAIN1), were identified as key regulators of the actin ring (figure 4). WAL is found to promote formation of the actin ring at the pit boundary to direct SCW deposition. BDR1 co-localizes, and directly interacts, with WAL at pit boundaries. BDR1 also interacts with the active form of ROP11, which then could be enriched at the SCW pits, providing a signalling cascade to the activating components of pit patterning. Indeed, it appears that ROP11 attracts BDR1 to the plasma membrane of the pits, which in turn recruits WAL [135].

Nevertheless, the mechanism by which BDR1 localize specifically at the boundary remains elusive. One possibility is that BDR1 first localizes evenly across the pit membrane, and then is excluded from the central region of pit membrane, thereby remaining only at the pit boundary. Another possibility is that other ROP members, which interacts with BDR1 more strongly than ROP11, are activated at the pit boundary to recruit BDR1. In summary, whereas a relatively detailed framework now is established for the patterning of the metaxylem SCWs, much remain to be explored regarding the mechanisms that drive protoxylem patterning. It appears likely that similar, or even the same, components are involved in both these processes; perhaps recruited and activated in different ways.

## 6. Conclusion and perspective

In the last decade, the processes of cell wall patterning in proto- and metaxylem vessel differentiation have been visualized largely thanks to the establishment of inducible xylem systems. For example, hormone- and VND6-inducible suspension cells have led us to identify key proteins and signalling pathways that regulate pitted cell wall patterns of metaxylem vessels. Studies on ROP signal pathways have revealed how the position and size of pits are determined and how the distinct structure of bordered pits is directed. Identification of IQD13 and CORD1 revealed regulatory mechanisms of pit shape. Still, much is unknown on the behaviour of these proteins and what factors regulate them. In addition to the VND6 inducible suspension cells, other systems, such as inducible hypocotyls or cotyledons, may help researchers to develop these mechanisms even further [88,134]. Analogously, the VND7 inducible system has largely been exploited in hypocotyl cells but could be further exploited in suspension cells or even protoplasts. Such approaches would increase the research portfolio and allow explorations whether, and how much, cell shape influences xylem patterns.

Though not discussed in this review, another important SCW-containing cell type is the xylary fibres. These cells are often characterized by having uniform deposition of SCWs with a few pits [200]. These cells provide the main structural support of angiosperms, allowing them to grow upright. Fibres are also the major cell type in the wood of dicots thus making them economically important. The master regulators of these cell types have been identified as the NAC transcription factors NST1, NST2 and NST3/SND1, which function together to control fibre development in arabidopsis [111–114]. Interestingly, ectopic overexpression of NST3/SND1 is sufficient to drive SCW development. However, the resulting walls do not have the characteristic fibre wall patterns. In fact, they sometimes produce helical SCW thickenings, similar to VND7, or pitted pattern, similar to VND6, despite not causing the upregulation of either of those transcription factors [112]. These data indicate that another yet to be identified regulatory mechanism controls SCW patterning in fibres, allowing them to form near uniform deposition of SCWs. Elucidation of such mechanisms represents a very important direction for future research which can have immediate economic benefits.

From the overwhelming experimental, theoretical and computational evidence gathered for the metaxylem patterns, it appears logical to apply this mechanism to explain other cell wall patterns, such as the strikingly ordered bands and coils present in protoxylem. However, the community has encountered a substantial challenge in doing so, since mutations in ROP11, ROPGAP3/4, ROPGEF4/7, IQD13 and -14, CORD1, BDR1 and WAL do not significantly impact the protoxylem wall. This indicates that periodic band patterning and pit formation are regulated by distinct mechanisms [131,134–136]. This raises the question of whether the protoxylem operates entirely differently from the metaxylem, or if there is a more complex gene redundancy underpinning protoxylem patterns. Additionally, much is known about how metaxylem patterns are regulated by Turing-like RD mechanisms where ROPs act as Turing morphogens, thereby controlling the pit patterns [201]. However, further research is needed to understand how this process is regulated in protoxylem. Another question that could be worthwhile asking is whether we can change a pattern into another at will, by altering ROP reaction–diffusion. For instance, Jacobs *et al*. [160] used partial differential equation models to study active ROP diffusion restriction as a mechanism of ROP pattern orientation in protoxylem development.

We currently know very little about the role of the lipid membrane in setting up ROP domain patterns. Whereas the formation of the metaxylem ROP pattern seems independent of lipid composition this may not be the case for other ROPs. By using fluorescent markers for plasma membrane ordering (e.g. ANEP dyes [202]) and lipid composition [203,204] the role of plasma membrane patterning can be unraveled. A systematic approach of testing all ROPs and GAP/GEFs (and combinations thereof) seems a logical additional step, particularly via CRISPR/Cas9-based strategies, allowing parallel knock-out of several potential candidate genes.

The consequences of altered cell wall patterns need to be linked to physiological traits. Are altered or dis-organized cell wall patterns detrimental for water conductivity or cavitation resistance? Are metaxylem (*gef4/7* and *gap3/4*, *iqd13*, *cord1*, etc.) and protoxylem (*pom2*, *ktn1-2*) wall pattering mutants more susceptible to water limiting conditions? This needs to be addressed for various model organisms ranging from thale cress over maize to poplar. Indeed, arabidopsis has been used to address questions related to the genetics of biophysics of water transport [205]. For example, recent studies in arabidopsis show that growing seedlings under reduced water potentials (using plates containing polyethylene glycol) causes the regular xylem architecture to be altered, with additional protoxylem cells and, interestingly, reticulate and protoxylem patterns in metaxylem cells appearing [206,207]. This offers a potential avenue to explore how a pitted wall pattern can be converted to a reticulated/band-like pattern within one cell type, particularly because the ability of metaxylem cells to make other wall patterns depended on VND7. Furthermore, similar cellular phenotypes were observed upon addition of abscisic acid (ABA) indicating a crucial role of this hormone in contributing to morphological responses under water limiting conditions. Interestingly, ABA-insensitive and ABA-synthesis mutants showed collapsed proto- and metaxylem vessels, a trait that can be rescued by the addition of ABA to the growth medium.

Another recent study in maize unraveled a protoxylem-specific gene—necrotic upper tips (NUT)1 belonging to the VND clade of arabidopsis—being important for water transport. The *nut1* phenotype (leaf wilting, necrosis, tassel browning, and sterility) is evident only after the floral transition (when water demand peaks) [208]. NUT1 localizes exclusively to developing protoxylem and its mutation causes thinner protoxylem vessels and compromised metaxylem cell wall integrity [208]. This is among the first studies showing that specifically protoxylem vessel integrity is linked with water transport defects.

Further upstream of the RD mechanism, small RNAs may mediate post-transcriptional modifications and epigenetic regulations during xylem development [209]. However, how these molecules affect SCW synthesis and patterning is yet to be fully defined. The function of miRNAs (micro RNAs) is to bind to mRNAs to hinder their expression or induce their degradation [210]. Many miRNAs were found to be up- or downregulated during SCW synthesis, targeting various proteins of different families, such as laccases, HD-Zip III transcription factors, superoxide dismutases and plantacyanins. Literature suggests that the SQUAMOSA PROMOTER-BINDING PROTEIN-LIKE transcription factor 7 (SPL7) is induced when the amount of available copper—which plays an important role in cell wall lignification—is limited. SPL7 thus induces the expression of copper proteins-targeting miRNAs, including miR397a, miR398b/c, miR408 and miR857, acting as a regulator of copper homeostasis [211]. Additionally, overexpression of miR166e and miR168a resulted in patterns with wider SCW bands spacing, suggesting that the two miRNAs are involved in SCW formation in protoxylem vessels. Therefore, it would be fascinating to further investigate the part that small RNAs play in SCW regulation.

Post-translational modifications of cell wall-related proteins also remains a topic in need of further exploration in relation to SCW patterning. Post-translational phosphorylation of primary wall CESAs *in vivo* changed the catalytic activity of CSC and its bidirectional velocities [212]. For instance, phospho-null mutation CESA1^S686A^ showed reduced root and hypocotyl length, and inconstant bi-directional movements were also observed in phospho-mutations [157]. However, phosphorylation in SCW CESAs is less understood except evidence from CESA7 phosphorylation *in vitro*, which suggested phosphorylation may destabilize SCW CESAs [213]. It could be that phosphorylation influences CSC behaviour and further impacts SCW patterns. In addition to phosphorylation, S-acylation were also identified to affect SCW banded patterns. Mutations of cysteines in variable region 2 and carboxy-terminus from CESA7 could traffic to Golgi but they were deficient in localization to the plasma localization and didn't show banded SCW patterns [25]. Post-translational modifications in non-CESAs proteins also affect SCW formation. SUMOylation of LBD30, which was mediated by SIZ1 in arabidopsis, regulates SCW formation in inflorescence fibre cells [214]. Moreover, N-glycosylation was also found to be required for the enzymatic activity of PtrMAN6 and suppresses SCW thickening in *Populus trichocarpa* [215,216]. Overall, it seems that post-translational modifications have an important effect on SCW formation, so it would be interesting to define how they change the patterning process.

As SCWs are thick and stiff, it is structurally difficult to degrade them for further saccharification, especially when it comes to lignin degradation. Recent research found that a primary-type wall could substitute SCW in fibre cells when expressing ERF035 under the control of NST3 promoter. This type of wall lacked lignin, but primary wall-related genes were activated [217]. The resulting xylem wall patterns were not investigated in these lines. However, this could be a good way to better understand how wall content may feedback onto pattern maintenance. These types of approaches may also form the platform to modify SCW structures and converting recalcitrant lignified SCWs into available walls for industrial products.

## Data Availability

This article has no additional data.
